# Diaqua­bis­(2-oxo-2*H*-chromene-3-carboxyl­ato)copper(II)

**DOI:** 10.1107/S1600536811018708

**Published:** 2011-05-20

**Authors:** Yue Cui, Qian Gao, Huan-Huan Wang, Lin Wang, Ya-Bo Xie

**Affiliations:** a College of Environmental and Energy Engineering, Beijing University of Technology, Beijing 100124, People’s Republic of China

## Abstract

In the title compound, [Cu(C_10_H_5_O_4_)_2_(H_2_O)_2_], the Cu^II^ atom lies on a crystallographic inversion center and exhibits an octa­hedral coordination defined by two O atoms from water mol­ecules in the axial positions and by four O atoms from two deprotonated coumarin-3-carb­oxy­lic acid ligands in the equatorial positions. The angles around the Cu^II^ atom vary between 85.32 (6) and 94.68 (6)°. The Cu—O bond distances between the Cu^II^ atom and the O atoms vary between 1.9424 (14) and 2.3229 (15) Å. The layers inter­digitate *via* face-to-face aromatic inter­actions [3.6490 (8) Å] between coumarin moieties such that the inter­layer separation is 10.460 (2) Å, *i.e.* the length of the *c* axis. O—H⋯O hydrogen bonds between the H atoms of coordinated water mol­ecules and the O atoms of carboxyl­ate groups link the complex mol­ecules into layers parallel to the *ab* plane.

## Related literature

For background to topological networks, see: Laza­rou *et al.* (2011 ▶). For applications of copper(II) complexes, see: Eddaoudi *et al.* (2001 ▶); Kirillov *et al.* (2010 ▶); Konidaris *et al.* (2009 ▶). For related structures, see: Wang *et al.* (2011 ▶).
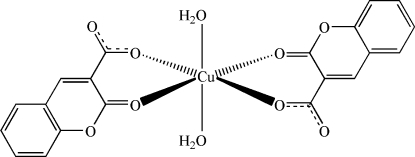


## Experimental

### 

#### Crystal data

[Cu(C_10_H_5_O_4_)_2_(H_2_O)_2_]
*M* *_r_* = 477.86
Triclinic, 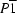

*a* = 6.5884 (13) Å
*b* = 6.8296 (14) Å
*c* = 10.460 (2) Å
α = 85.98 (3)°
β = 89.79 (3)°
γ = 65.38 (3)°
*V* = 426.65 (15) Å^3^
*Z* = 1
Mo *K*α radiation
μ = 1.35 mm^−1^
*T* = 293 K
0.20 × 0.15 × 0.15 mm

#### Data collection

Bruker APEXII CCD diffractometer
Absorption correction: multi-scan (*SADABS*; Sheldrick, 2008*a* ▶) *T*_min_ = 0.785, *T*_max_ = 0.817
2696 measured reflections
1954 independent reflections
1926 reflections with *I* > 2σ(*I*)
*R*_int_ = 0.014

#### Refinement

*R*[*F* ^2^ > 2σ(*F* ^2^)] = 0.026
*wR*(*F* ^2^) = 0.080
*S* = 1.10
1954 reflections
146 parameters
H atoms treated by a mixture of independent and constrained refinement
Δρ_max_ = 0.47 e Å^−3^
Δρ_min_ = −0.59 e Å^−3^

### 

Data collection: *APEX2* (Bruker, 2008 ▶); cell refinement: *SAINT* (Bruker, 2008 ▶); data reduction: *SAINT*; program(s) used to solve structure: *SHELXS97* (Sheldrick, 2008*b* ▶); program(s) used to refine structure: *SHELXL97* (Sheldrick, 2008*b* ▶); molecular graphics: *SHELXTL* (Sheldrick, 2008*b* ▶); software used to prepare material for publication: *SHELXTL*.

## Supplementary Material

Crystal structure: contains datablocks global, I. DOI: 10.1107/S1600536811018708/zk2008sup1.cif

Structure factors: contains datablocks I. DOI: 10.1107/S1600536811018708/zk2008Isup2.hkl

Additional supplementary materials:  crystallographic information; 3D view; checkCIF report

## Figures and Tables

**Table 1:** Hydrogen-bond geometry (Å, °)

*D*—H⋯*A*	*D*—H	H⋯*A*	*D*⋯*A*	*D*—H⋯*A*
O1*W*—H1*WA*⋯O4^i^	0.82	1.89	2.706 (2)	177
O1*W*—H1*WB*⋯O4^ii^	0.88 (3)	1.90 (3)	2.753 (2)	163 (3)

Symmetry codes: (i) 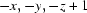; (ii) 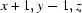.

**Fig. 1.**
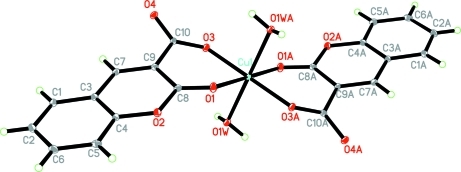
The molecular structure of the title compound with displacement ellipsoids drawn at the 50% probability level for non-hydrogen atoms, hydrogen atoms are shown as small circles of arbitrary radius. [Symmetry code: i = -x + 1, -y, -z + 1].

**Fig. 2.**
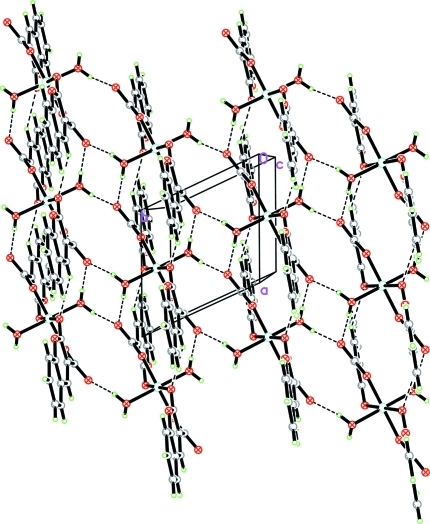
Partial packing view of title compound, showing the formation of network built from hydrogen bonds.

## supplementary crystallographic information

### Comment

In the past decades, numerous papers dealing with copper(II) complexes have been published due to their fascinating structural diversity (Lazarou *et al.*, 2011) and potential applications in the areas of catalysis (Kirillov *et al.*, 2010), gas addsorption (Eddaoudi *et al.*, 2001), magnetism (Konidaris *et al.*, 2009) and so on. Herein, we report the synthesis and crystal structure of a new mononuclear copper complex coordinated by coumarin-3-carboxylic acid.

In the title compound, [Cu(C_10_H_5_O_4_)_2_(H_2_O)_2_], copper(II) atom lies on a crystallographic inversion center and exhibits octahedral geometry with the coordination of two O atoms from water molecules in the axial positions and four O atoms from two deprotonated coumarin-3-carboxylic acid ligands in the equatorial positions. Angles around the Cu^II^ atom vary between 85.32 (6)° and 94.68 (6)°. The Cu—O bond distances between the Cu^II^ atom and the O atoms vary between 1.9424 (14) and 2.3229 (15) Å, all of which are comparable to those reported for other copper-oxygen donor complexes (*e.g.*, Wang *et al.*, 2011). The (C2C1C3C4C5C6) ring and the (C4C3C7C9C8O2) ring are almost coplanar, and the dihedral angles is 1.568 (57)°. The layers interdigitate *via* face to face aromatic interactions (distance 3.6490 (8) Å) between coumarin moieties such that the interlayer separation is 10.46 Å, length of *c* axis. O—H···O hydrogen bonds between the hydrogen atoms of coordinated water molecules and the O atoms of carboxylate groups joins the complexes into two-dimensional layers parallel the *ab* plane (Table 1 and Fig. 2).

### Experimental

The title complex was synthesized by carefully layering a solution of Cu(NO_3_)_2_.3H_2_O (24.2 mg, 0.1 mmol) in ethanol (10 ml) on top of a solution of coumarin-3-carboxylic acid (19.0 mg, 0.1 mmol) and LiOH (8.4 mg, 0.2 mmol) in H_2_O (10 ml) in a test-tube. After about one month at room temperature, green block-shaped single crystals suitable for X-ray investigation appeared at the boundary between the ethanol solution and the water layer with a yield of 25% (12.1 mg). FT—IR (KBr, cm^-1^): 788, 1028, 1183, 1285, 1388, 1457, 1560, 1697, 3180.

### Refinement

Carbon H atoms were placed geometrically (C—H = 0.93 Å) and treated as riding with *U*_iso(H)_ = 1.2*U*_eq_(C). Water H atoms were located in calculated positions and treated in the subsequent refinement as riding atoms, with O—H = 0.85 Å and *U*_iso_(H) = 1.5*U*_eq_(O).

### Crystal data

**Table:**

[Cu(C_10_H_5_O_4_)_2_(H_2_O)_2_]	*Z* = 1
*M**_r_* = 477.86	*F*(000) = 243
Triclinic, *P*1	*D*_x_ = 1.860 Mg m^−^^3^
Hall symbol: -P 1	Mo *K*α radiation, λ = 0.71073 Å
*a* = 6.5884 (13) Å	Cell parameters from 2273 reflections
*b* = 6.8296 (14) Å	θ = 3.3–28.3°
*c* = 10.460 (2) Å	µ = 1.35 mm^−^^1^
α = 85.98 (3)°	*T* = 293 K
β = 89.79 (3)°	Block, green
γ = 65.38 (3)°	0.20 × 0.15 × 0.15 mm
*V* = 426.65 (15) Å^3^	

### Data collection

**Table:**

Bruker APEXII CCD diffractometer	1954 independent reflections
Radiation source: fine-focus sealed tube	1926 reflections with *I* > 2σ(*I*)
graphite	*R*_int_ = 0.014
φ and ω scans	θ_max_ = 28.3°, θ_min_ = 3.3°
Absorption correction: multi-scan (*SADABS*; Sheldrick, 2008*a*)	*h* = −8→8
*T*_min_ = 0.785, *T*_max_ = 0.817	*k* = −6→8
2696 measured reflections	*l* = −12→13

### Refinement

**Table:**

Refinement on *F*^2^	Primary atom site location: structure-invariant direct methods
Least-squares matrix: full	Secondary atom site location: difference Fourier map
*R*[*F*^2^ > 2σ(*F*^2^)] = 0.026	Hydrogen site location: inferred from neighbouring sites
*wR*(*F*^2^) = 0.080	H atoms treated by a mixture of independent and constrained refinement
*S* = 1.10	*w* = 1/[σ^2^(*F*_o_^2^) + (0.0426*P*)^2^ + 0.4127*P*] where *P* = (*F*_o_^2^ + 2*F*_c_^2^)/3
1954 reflections	(Δ/σ)_max_ < 0.001
146 parameters	Δρ_max_ = 0.47 e Å^−^^3^
0 restraints	Δρ_min_ = −0.59 e Å^−^^3^

### Special details

**Table:**

Geometry. All e.s.d.'s (except the e.s.d. in the dihedral angle between two l.s. planes) are estimated using the full covariance matrix. The cell e.s.d.'s are taken into account individually in the estimation of e.s.d.'s in distances, angles and torsion angles; correlations between e.s.d.'s in cell parameters are only used when they are defined by crystal symmetry. An approximate (isotropic) treatment of cell e.s.d.'s is used for estimating e.s.d.'s involving l.s. planes.
Refinement. Refinement of *F*^2^ against ALL reflections. The weighted *R*-factor *wR* and goodness of fit *S* are based on *F*^2^, conventional *R*-factors *R* are based on *F*, with *F* set to zero for negative *F*^2^. The threshold expression of *F*^2^ > σ(*F*^2^) is used only for calculating *R*-factors(gt) *etc*. and is not relevant to the choice of reflections for refinement. *R*-factors based on *F*^2^ are statistically about twice as large as those based on *F*, and *R*- factors based on ALL data will be even larger.

### Fractional atomic coordinates and isotropic or equivalent isotropic displacement parameters (Å^2^)

**Table:**

	*x*	*y*	*z*	*U*_iso_*/*U*_eq_	
Cu1	0.5000	0.0000	0.5000	0.00966 (11)	
O1	0.4519 (2)	0.1657 (2)	0.69122 (12)	0.0146 (3)	
O1W	0.4903 (2)	−0.2639 (2)	0.59038 (12)	0.0126 (3)	
H1WA	0.3929	−0.2900	0.5568	0.019*	
O3	0.1764 (2)	0.1397 (2)	0.48277 (12)	0.0124 (3)	
O4	−0.1736 (2)	0.3644 (2)	0.51356 (12)	0.0127 (3)	
O2	0.3051 (2)	0.1949 (2)	0.88105 (12)	0.0122 (3)	
C1	−0.2504 (3)	0.2859 (3)	1.00004 (18)	0.0135 (3)	
H1A	−0.3904	0.3043	0.9699	0.016*	
C2	−0.2116 (3)	0.2857 (3)	1.13001 (18)	0.0155 (4)	
H2A	−0.3264	0.3068	1.1869	0.019*	
C3	−0.0782 (3)	0.2581 (3)	0.91346 (17)	0.0112 (3)	
C4	0.1306 (3)	0.2267 (3)	0.96207 (17)	0.0113 (3)	
C5	0.1731 (3)	0.2237 (3)	1.09260 (17)	0.0139 (3)	
H5A	0.3138	0.2019	1.1231	0.017*	
C6	0.0000 (3)	0.2539 (3)	1.17600 (17)	0.0154 (4)	
H6A	0.0249	0.2530	1.2635	0.019*	
C7	−0.1071 (3)	0.2648 (3)	0.77726 (17)	0.0111 (3)	
H7A	−0.2457	0.2871	0.7429	0.013*	
C8	0.2831 (3)	0.1975 (3)	0.75042 (17)	0.0110 (3)	
C9	0.0633 (3)	0.2392 (3)	0.69730 (17)	0.0103 (3)	
C10	0.0214 (3)	0.2495 (3)	0.55452 (17)	0.0103 (3)	
H1WB	0.614 (5)	−0.377 (5)	0.577 (3)	0.035 (8)*	

### Atomic displacement parameters (Å^2^)

**Table:**

	*U*^11^	*U*^22^	*U*^33^	*U*^12^	*U*^13^	*U*^23^
Cu1	0.00616 (16)	0.01127 (16)	0.00921 (16)	−0.00141 (11)	0.00012 (10)	−0.00028 (11)
O1	0.0097 (6)	0.0224 (7)	0.0120 (6)	−0.0065 (5)	0.0005 (5)	−0.0029 (5)
O1W	0.0094 (6)	0.0135 (6)	0.0130 (6)	−0.0029 (5)	−0.0002 (5)	−0.0003 (5)
O3	0.0089 (6)	0.0157 (6)	0.0103 (6)	−0.0025 (5)	−0.0001 (5)	−0.0018 (5)
O4	0.0086 (6)	0.0143 (6)	0.0127 (6)	−0.0022 (5)	−0.0018 (5)	−0.0006 (5)
O2	0.0096 (6)	0.0166 (6)	0.0098 (6)	−0.0050 (5)	−0.0007 (5)	−0.0010 (5)
C1	0.0118 (8)	0.0129 (8)	0.0143 (8)	−0.0039 (6)	0.0025 (7)	−0.0001 (6)
C2	0.0183 (9)	0.0130 (8)	0.0132 (8)	−0.0046 (7)	0.0052 (7)	−0.0004 (6)
C3	0.0112 (8)	0.0095 (7)	0.0113 (8)	−0.0029 (6)	0.0010 (6)	−0.0004 (6)
C4	0.0121 (8)	0.0096 (7)	0.0106 (8)	−0.0030 (6)	0.0017 (6)	−0.0007 (6)
C5	0.0157 (9)	0.0124 (8)	0.0120 (8)	−0.0044 (7)	−0.0032 (7)	0.0000 (6)
C6	0.0221 (10)	0.0123 (8)	0.0097 (8)	−0.0050 (7)	0.0005 (7)	−0.0005 (6)
C7	0.0094 (8)	0.0104 (8)	0.0122 (8)	−0.0031 (6)	−0.0005 (6)	−0.0003 (6)
C8	0.0113 (8)	0.0102 (7)	0.0103 (8)	−0.0033 (6)	−0.0012 (6)	−0.0006 (6)
C9	0.0091 (8)	0.0100 (7)	0.0107 (8)	−0.0030 (6)	−0.0009 (6)	−0.0005 (6)
C10	0.0092 (8)	0.0099 (7)	0.0117 (8)	−0.0039 (6)	0.0007 (6)	−0.0001 (6)

### Geometric parameters (Å, °)

**Table:**

Cu1—O3^i^	1.9424 (14)	C1—C3	1.408 (2)
Cu1—O3	1.9424 (14)	C1—H1A	0.9300
Cu1—O1W^i^	2.0007 (14)	C2—C6	1.400 (3)
Cu1—O1W	2.0007 (14)	C2—H2A	0.9300
Cu1—O1	2.3229 (15)	C3—C4	1.393 (3)
Cu1—O1^i^	2.3229 (15)	C3—C7	1.433 (2)
O1—C8	1.216 (2)	C4—C5	1.392 (2)
O1W—H1WA	0.8200	C5—C6	1.388 (3)
O1W—H1WB	0.88 (3)	C5—H5A	0.9300
O3—C10	1.266 (2)	C6—H6A	0.9300
O4—C10	1.251 (2)	C7—C9	1.357 (2)
O2—C8	1.373 (2)	C7—H7A	0.9300
O2—C4	1.377 (2)	C8—C9	1.459 (2)
C1—C2	1.384 (3)	C9—C10	1.511 (2)
			
O3^i^—Cu1—O3	180.0	C6—C2—H2A	119.9
O3^i^—Cu1—O1W^i^	91.47 (6)	C4—C3—C1	118.49 (16)
O3—Cu1—O1W^i^	88.53 (6)	C4—C3—C7	118.02 (16)
O3^i^—Cu1—O1W	88.53 (6)	C1—C3—C7	123.47 (17)
O3—Cu1—O1W	91.47 (6)	O2—C4—C5	117.36 (16)
O1W^i^—Cu1—O1W	180.0	O2—C4—C3	120.44 (16)
O3^i^—Cu1—O1	94.68 (6)	C5—C4—C3	122.19 (17)
O3—Cu1—O1	85.32 (6)	C6—C5—C4	118.29 (18)
O1W^i^—Cu1—O1	88.76 (6)	C6—C5—H5A	120.9
O1W—Cu1—O1	91.24 (6)	C4—C5—H5A	120.9
O3^i^—Cu1—O1^i^	85.32 (6)	C5—C6—C2	120.83 (17)
O3—Cu1—O1^i^	94.68 (6)	C5—C6—H6A	119.6
O1W^i^—Cu1—O1^i^	91.24 (6)	C2—C6—H6A	119.6
O1W—Cu1—O1^i^	88.76 (6)	C9—C7—C3	121.45 (17)
O1—Cu1—O1^i^	180.0	C9—C7—H7A	119.3
C8—O1—Cu1	118.65 (12)	C3—C7—H7A	119.3
Cu1—O1W—H1WA	109.5	O1—C8—O2	115.47 (16)
Cu1—O1W—H1WB	110 (2)	O1—C8—C9	127.02 (16)
H1WA—O1W—H1WB	103.7	O2—C8—C9	117.51 (15)
C10—O3—Cu1	134.40 (12)	C7—C9—C8	119.69 (16)
C8—O2—C4	122.81 (14)	C7—C9—C10	118.84 (16)
C2—C1—C3	120.02 (18)	C8—C9—C10	121.45 (15)
C2—C1—H1A	120.0	O4—C10—O3	122.88 (16)
C3—C1—H1A	120.0	O4—C10—C9	116.53 (15)
C1—C2—C6	120.16 (17)	O3—C10—C9	120.53 (16)
C1—C2—H2A	119.9		
			
O3^i^—Cu1—O1—C8	−149.14 (14)	C1—C2—C6—C5	0.5 (3)
O3—Cu1—O1—C8	30.86 (14)	C4—C3—C7—C9	0.7 (3)
O1W^i^—Cu1—O1—C8	119.49 (14)	C1—C3—C7—C9	179.37 (17)
O1W—Cu1—O1—C8	−60.51 (14)	Cu1—O1—C8—O2	149.88 (11)
O1W^i^—Cu1—O3—C10	−95.25 (17)	Cu1—O1—C8—C9	−30.6 (2)
O1W—Cu1—O3—C10	84.75 (17)	C4—O2—C8—O1	−179.60 (15)
O1—Cu1—O3—C10	−6.37 (17)	C4—O2—C8—C9	0.9 (2)
O1^i^—Cu1—O3—C10	173.63 (17)	C3—C7—C9—C8	1.8 (3)
C3—C1—C2—C6	−1.2 (3)	C3—C7—C9—C10	−179.55 (15)
C2—C1—C3—C4	1.2 (3)	O1—C8—C9—C7	177.91 (18)
C2—C1—C3—C7	−177.46 (16)	O2—C8—C9—C7	−2.6 (2)
C8—O2—C4—C5	−179.00 (15)	O1—C8—C9—C10	−0.7 (3)
C8—O2—C4—C3	1.7 (3)	O2—C8—C9—C10	178.83 (15)
C1—C3—C4—O2	178.78 (15)	Cu1—O3—C10—O4	163.62 (13)
C7—C3—C4—O2	−2.5 (3)	Cu1—O3—C10—C9	−19.1 (3)
C1—C3—C4—C5	−0.5 (3)	C7—C9—C10—O4	28.9 (2)
C7—C3—C4—C5	178.26 (16)	C8—C9—C10—O4	−152.53 (17)
O2—C4—C5—C6	−179.54 (16)	C7—C9—C10—O3	−148.55 (17)
C3—C4—C5—C6	−0.2 (3)	C8—C9—C10—O3	30.0 (2)
C4—C5—C6—C2	0.3 (3)		

Symmetry codes: (i) −*x*+1, −*y*, −*z*+1.

### Hydrogen-bond geometry (Å, °)

**Table:**

*D*—H···*A*	*D*—H	H···*A*	*D*···*A*	*D*—H···*A*
O1W—H1WA···O4^ii^	0.82	1.89	2.706 (2)	177
O1W—H1WB···O4^iii^	0.88 (3)	1.90 (3)	2.753 (2)	163 (3)

Symmetry codes: (ii) −*x*, −*y*, −*z*+1; (iii) *x*+1, *y*−1, *z*.
